# Induction of sexual reproduction and genetic diversity in the cheese fungus *Penicillium roqueforti*

**DOI:** 10.1111/eva.12140

**Published:** 2014-03-20

**Authors:** Jeanne Ropars, Manuela López-Villavicencio, Joëlle Dupont, Alodie Snirc, Guillaume Gillot, Monika Coton, Jean-Luc Jany, Emmanuel Coton, Tatiana Giraud

**Affiliations:** 1 Ecologie, Systématique et Evolution, Université Paris-SudOrsay Cedex, France; 2 CNRSOrsay Cedex, France; 3 Origine, Structure, Evolution de la Biodiversité, UMR 7205 CNRS-MNHN, Muséum National d'Histoire NaturelleParis Cedex 05, France; 4 Université de Brest, EA 3882, Laboratoire Universitaire de Biodiversité et d'Ecologie Microbienne, ESIAB, Technopôle Brest-IroisePlouzané, France

**Keywords:** *Aspergillus*, domestication, HGT, population structure, sex, wild strains, yeast

## Abstract

The emblematic fungus *Penicillium roqueforti* is used throughout the world as a starter culture in the production of blue-veined cheeses. Like other industrial filamentous fungi, *P. roqueforti* was thought to lack a sexual cycle. However, an ability to induce recombination is of great economic and fundamental importance, as it would make it possible to transform and improve industrial strains, promoting the creation of novel phenotypes and eliminating the deleterious mutations that accumulate during clonal propagation. We report here, for the first time, the induction of the sexual structures of *P. roqueforti* — ascogonia, cleistothecia and ascospores. The progeny of the sexual cycle displayed clear evidence of recombination. We also used the recently published genome sequence for this species to develop microsatellite markers for investigating the footprints of recombination and population structure in a large collection of isolates from around the world and from different environments. Indeed, *P. roqueforti* also occurs in silage, wood and human-related environments other than cheese. We found tremendous genetic diversity within *P. roqueforti*, even within cheese strains and identified six highly differentiated clusters that probably predate the use of this species for cheese production. Screening for phenotypic and metabolic differences between these populations could guide future development strategies.

## Introduction

Cheese is an important foodstuff introduced into the human diet in the early Neolithic Era (Beresford et al. 2001; Salque et al. 2013). Cheese-making increases the quality and duration of milk conservation and the digestibility of lactose (Bennett and Johnston 2004). Two categories of microorganisms are required for cheese-making: primary and secondary flora (Salque et al. 2013). The primary flora consists of lactic acid bacteria, which have been studied in detail (Mounier et al. 2008; Irlinger and Mounier 2009; Douglas and Klaenhammer 2010). The secondary flora develops after the environment has been modified by the primary flora and consists of a mixture of bacteria, yeasts and moulds playing an important role in the texture and flavour of cheeses.

*Penicillium roqueforti* is used in the production of the French cheese Roquefort and in many other blue-veined cheeses around the world, including Italian gorgonzola, Danablue in Denmark, Stilton in United Kingdom and Cabrales in Spain. This species is therefore of great economic importance. In France alone, the total production of Roquefort and other blue cheeses reached almost 57 000 tonnes in 2011 (Martin-Houssart 2012). Indeed, Roquefort cheese is one of the most emblematic cheeses in France, produced and exported in large amounts, and it was the first cheese to obtain the ‘Appellation d'Origine Controlée’ (AOC) label, which guarantees the origin of traditional French products. By contrast to many other industrial species, such as *P. camemberti,* wild strains of *P. roqueforti* can be found, allowing comparisons between industrial and wild strains for understanding the domestication process and history. In addition to its presence in dairy environments, *P. roqueforti* is a common spoilage agent in refrigerated stored foods, meat, wheat products and silage, and it is even found in forest soil and wood (Pitt and Hocking 2009).

*Penicillium roqueforti*, like other industrial filamentous fungi and an estimated one-fifth of fungal species, was thought to lack a sexual cycle. However, evidence for the occurrence of sexual reproduction has been obtained for many of the supposed ‘asexual’ species (Burt et al. 1996; Pringle et al. 2005; Rydholm et al. 2006), and indirect evidence of sexual reproduction has recently been reported for *P. roqueforti* (Ropars et al. 2012). Mating type genes (MAT genes), which are involved in gamete recognition and mating in fungi, and other genes involved in meiosis, have been detected in the *P. roqueforti* genome and appear to be subject to strong purifying selection, suggesting that they are still used and functional. Haploid strains of *P. roqueforti* carry a single allele of the MAT gene in their genome, as all heterothallic fungi, meaning that fusion is possible only between gametes with opposing MAT alleles. Population analyses with polymorphic markers have revealed footprints of recombination and both MAT alleles have been detected. Other indirect evidence for the occurrence of sex in *P. roqueforti* includes the detection of RIP (repeat-induced point mutation) footprints (Ropars et al. 2012), a phenomenon in which repeated sequences are mutated and which occurs in fungi exclusively during the dikaryotic phase between syngamy and meiosis.

However, sexual structures have never been observed in *P. roqueforti,* in either natural or controlled conditions. Industrial strains are maintained by ferment producers and are replicated clonally. As for other industrial species, efforts to identify novel traits have thus been based purely on random mutagenesis and screening (Pöggeler 2001). The possibility of inducing a sexual cycle would be of considerable interest for industrial purposes, as it would facilitate the generation, by recombination, and selection of interesting new traits, in terms of colour, growth rate and metabolite production, for example. It would also make it possible to correct and prevent the degeneration generally observed in clonal species (Pöggeler 2001; Bruggeman et al. 2003). Sexual cycles have recently been successfully triggered in laboratory conditions in close relatives of *P. roqueforti,* such as *Aspergillus fumigatus* (O'Gorman et al. 2009), *A. lentulus* (Swilaiman et al. 2013) and *P. chrysogenum* (Böhm et al. 2012).

Here, we report the successful induction of a sexual cycle in *P. roqueforti*, using individuals from different environments that had previously been screened for mating type (Ropars et al. 2012). We also used the recently published genome sequence for this species (Cheeseman et al. 2014) to develop microsatellite markers and found recombination footprints, a tremendous diversity and a strong population structure in a large collection of 114 isolates from different environments and locations (Table S1).

## Materials and methods

### Fungal isolates

We used 114 strains of *P. roqueforti* described in a previous study (Ropars et al. 2012) (Table S1). All these strains are kept in the collection of the Natural History Museum in Paris or the LUBEM collection. The fungal strains were of industrial origin or obtained from silage and natural habitats. Single-spore isolation was systematically carried out by a dilution method to guarantee that colonies originated from a single genotype.

### DNA extraction

Genomic DNA was extracted from fresh mycelium of the isolates listed in Table S1 grown for 3–5 days on malt agar. The Qiagen DNeasy Plant Mini Kit (Qiagen, Ltd. Crawley, UK) was used for DNA extraction and purification. We used the 5′ end of the *β*-tubulin gene (oligonucleotide primer set Bt2a/Bt2b; Glass and Donaldson 1995) to type strains, to ensure that they belonged to the *P. roqueforti* species (Samson et al. 2004).

### Induction of sex

Eight *P. roqueforti* isolates were crossed in all possible pairwise MAT1-1 (four strains) and MAT1-2 (four strains) combinations, in duplicate, following the protocol recently published for *P. chrysogenum* (Böhm et al. 2012). Two different media culture were used, oatmeal agar (Böhm et al. 2012) and goat-cheese agar media (Decker and Nielsen 2005), both supplemented with biotin (6.4 μg/L) after autoclaving (Böhm et al. 2012). Petri dishes were sealed with Parafilm and incubated at 15 or 25°C in the dark. For each isolate, spore suspensions containing 1 × 10^5^ conidia per mL were prepared (O'Gorman et al. 2009), from seven-day-old cultures. On each plate, 5 μL of a given spore suspension was used to inoculate sections of agar at opposite sites of the plate, located perpendicular to sections inoculated with aliquots of conidia of the opposite mating type (O'Gorman et al. 2009). Crosses were examined with a binocular loupe and an optical microscope, to check for cleistothecia, periodically over a period of 4 weeks. Photographs were taken with a Nikon D300 camera, on a luminescence microscope (Nikon France, Champigny-sur-Marne, France).

### Recombination and segregation in the progeny

For genotyping the sexual progeny, we tried to recover ascospores by rolling cleistothecia as previously described (O'Gorman et al. 2009). This protocol, however, failed to eliminate enough conidia because the thin walls of the cleistothecia made their manipulation difficult. We therefore tried eliminating the asexual conidia surrounding the cleistothecia, by heating (Houbraken et al. 2008). We tested the resistance of the conidia to heating by placing suspensions of conidia in water in a dry heat bain-marie at different temperatures, ranging from 30 to 90°C for 5, 10 and 15 min. Suspensions were then plated on malt agar and incubated for 3 days at 25°C, and the colonies formed were counted. This experiment revealed that conidia survived heating for up to 10 min at temperatures below 80°C. No growth was observed for conidia heated at 80, 85 and 90°C. We then picked several cleistothecia from the original Petri dishes of the LCP 4111 × LCP 3914 crosses with a needle and suspended them in 1 mL of sterilized water supplemented with Tween. The cleistothecia were then heated at 80 and 85°C for 10 and 15 min to eliminate conidia. Heated suspensions were plated on malt agar and incubated for 5 days, after the cleistothecia had been ground. The colonies growing at high temperatures were presumed to result exclusively from sexual ascospore germination. Single-spore isolation was carried out with a dilution method, to guarantee the presence of a single individual on malt agar Petri dishes. After 5 days of growth at 25°C, seven individuals were picked and plated on other Petri dishes for growth at 25°C, for DNA extraction and segregation tests.

### Population genetics analyses

#### Design of polymorphic microsatellite markers

We looked for microsatellite motifs in the genomic sequence of FM164 (Cheeseman et al. 2014) and designed 94 primer pairs binding to the regions flanking these motifs (Table S2). We selected the polymorphic markers in a multiplex analysis (Cryer et al. 2005). Amplifications were performed in a Bio-Rad (Marnes-la-Coquette, France) DNA Engine Peltier Thermal cycler using a touchdown program with 35 cycles of 30 s at 94°C, a decreasing of 1°C every 30 s from 60 to 50°C, and 60 s at 72°C. The PCR program was followed by a final 7 min extension step at 72°C.

Using six isolates for a first screening of polymorphism, we pooled unlabelled PCR amplicons and ligated them into standard cloning vectors. PCR was then performed with a labelled universal primer binding to the region flanking the plasmid insert and unlabelled locus-specific primers. The previously used touchdown programme was reused for DNA amplification. Electrophoresis genotyping by capillary fractionation was carried out at INRA Clermont-Ferrand (*Plateforme Stratégique INRA*, Ibisa 2009, ISO9001:2008). The profiles obtained for the six isolates were analysed with genemapper Software Version 4.0 (Applied Biosystem, Villebon-sur-Yvette, France). This led to the selection of 11 polymorphic loci, *that is,* loci at which two different alleles (Table S2) were identified in the six isolates tested. The physical independence of these 11 loci was checked by their localization within the reference genome (see scaffold names in Table S2). The whole collection was then screened using these 11 polymorphic microsatellite markers. Only a few ascospores were genotyped, with a subset of the markers, as only a few recombination events between parental genotypes were required to show that the ascospores actually resulted from meioses.

### Genetic analyses

A splitstree was inferred with splitstree 4 software (Huson and Bryant 2006) (http://www.splitstree.org/). Factorial correspondence analyses (FCA) were performed with genetix v4.05 (Belkhir et al. 1996). Linkage disequilibrium among the 11 markers and population differentiation were assessed with genepop on the Web (Raymond and Rousset 1995) (http://genepop.curtin.edu.au/).

We used the individual-based Bayesian clustering methods implemented in STRUCTURE 2.3.3 (Pritchard et al. 2000) to infer population structure. STRUCTURE makes use of Markov Chain Monte Carlo (MCMC) simulations to infer the proportion of ancestry of genotypes from *K* distinct clusters. The underlying algorithms attempt to minimize deviations from Hardy–Weinberg and linkage disequilibria. Ten independent analyses were carried out for each number of clusters, from *K* = 2 to *K* = 10, using admixture models and 500 000 MCMC iterations, after a burn in of 50 000 steps. The output was processed using clumpp v1.1.2 (Jakobsson and Rosenberg 2007), to identify clustering solutions in replicated runs for each value of *K*. Population structure was then displayed graphically with distruct v1.1 (Rosenberg 2004). We implemented the Evanno method (Evanno et al. 2005) via the Structure Harvester website (Earl and VonHoldt 2012) (http://taylor0.biology.ucla.edu/structureHarvester/), to identify the *K* value corresponding to the strongest structure.

## Results

### Induction of sexual reproduction

**Figure 1:**
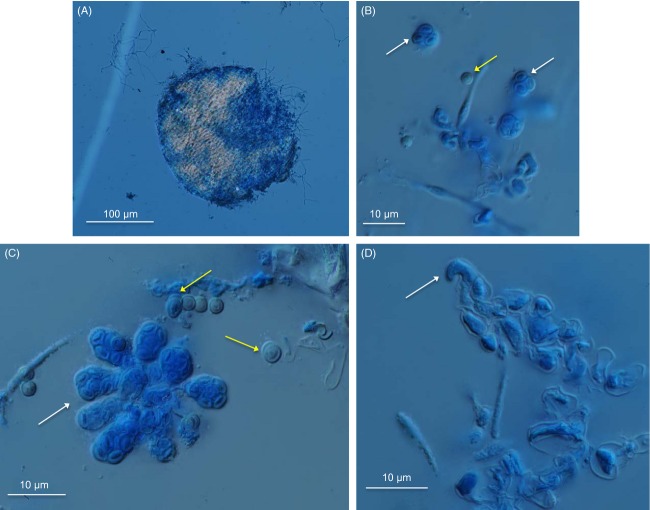
Sexual structures of *Penicillium roqueforti*. (A) One cleistothecium. (B–C) White arrows show asci-containing ascospores (sexual spores), whereas yellow ones show conidia (asexual spore). (D) Ascogonia (female sexual structures).

Twelve crosses between individuals previously screened for mating type and isolated from industrial and ‘natural’ environments (i.e. noncheese environments) were grown on biotin-supplemented oatmeal medium or biotin-supplemented cheese medium at 25 or 15°C, in the dark. After 3 weeks of culture, nine of the crosses growing on oatmeal medium at 25°C produced fruiting bodies (cleisthothecia), which, for one of these crosses [LCP 03914 (MAT1-1) × LCP 04111 (MAT1-2)], contained mature asci with ascospores (Fig. 1). In this cross, we were also able to observe female sexual structures*,* ascogonia (Fig. 1D, Table 1A). Cleistothecia were also formed on oatmeal medium at 15°C, but no mature ascospores could be isolated from fungi grown at this temperature. Cleistothecia failed to develop on cheese medium.

**Table 1:** Induction of sex in *Penicillium roqueforti*. (A) Results of 16 crosses tested for sex induction in *Penicillium roqueforti*. A ‘x’ indicates that no sexual structure was observed. (B) Evidence of recombination in *Penicillium roqueforti* ascospores isolated from cleistothecia obtained in the LCP 03914 × LCP 04111 cross.

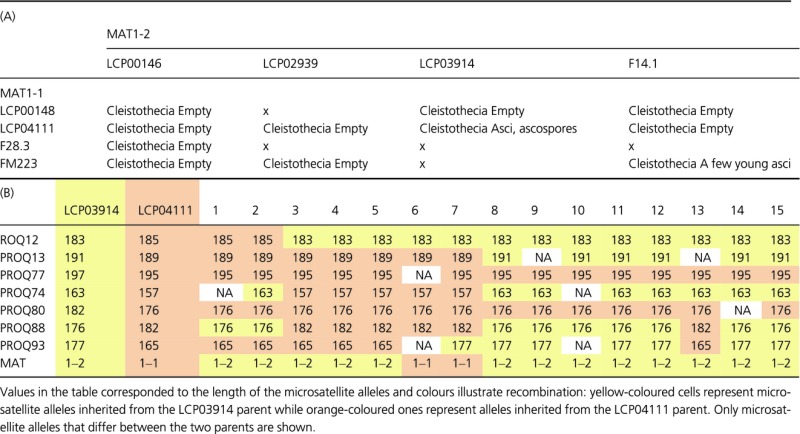

We checked for recombination, by isolating seven ascospores from mature LPC 03914 × LCP 04111 asci and investigating segregation at 11 microsatellite markers for which alleles differed between the parental strains and at loci for mating type genes. We found clear evidence of recombination in the progeny (Table 1B).

### Genetic diversity and population structure in *Penicillium roqueforti*

**Figure 2:**
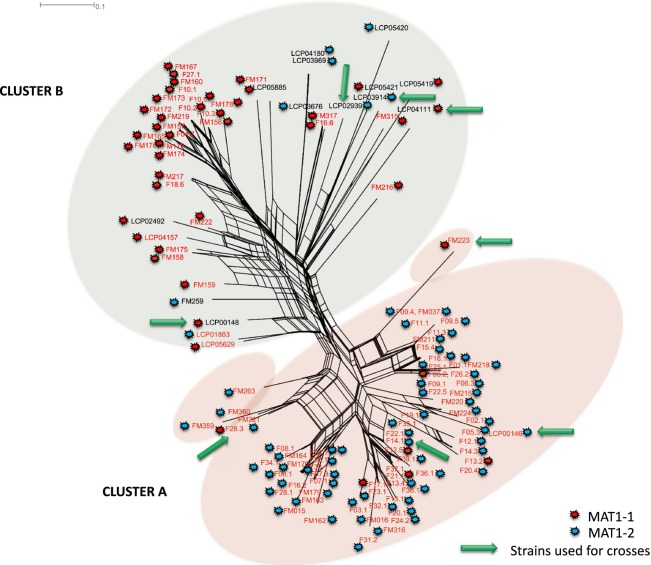
Splitstree of *Penicillium roqueforti*. Splitstree4 diagram for 114 *Penicillium roqueforti* strains from different collections and isolated from cheeses worldwide based on 11 microsatellite locus. Reticulation indicates likely occurrence of recombination.

A large collection of *P. roqueforti* isolates (*N *=* *114) was genotyped. Isolates were collected from dairy environments (*N *=* *101), silage (*N *=* *2), wood (*N *=* *1) or other noncheese environments, such as stewed fruit, the inside wall of a fridge, brioche packaging or the atmosphere of a brewery (*N *=* *10). A Splitstree and a NJ tree were constructed from the distance matrix generated from the 11 microsatellite markers (Fig. 2 and Figure S1). Footprints of clonal propagation were found in industrial isolates, with some isolates having identical genotypes for all 11 markers (Fig. 2, several F and FM strains, in red, on the same tip). By contrast, no isolates belonging to the same clone were observed among noncheese isolates (Fig. 2, noncheese strains, represented in black, on different branches).

The trees revealed the existence of two clusters (Fig. 2 and Figure S1), which encompassed strains with contrasted features and origins. The A cluster contained only cheese strains, most of which carried *Wallaby*, a large genomic region (ca. 500 kb), which was recently shown to have been transferred horizontally between different *Penicillium* species from the cheese environment (Cheeseman et al. 2014). There were four exceptions, the industrial ‘F’ strains F07.1, F09.5, F13.1 and F22.5, in which *Wallaby* was not detected with our primer pairs (Cheeseman et al. 2014). By contrast, cluster B included strains isolated from diverse environments, such as wood, silage and cheese, and none of the strains of this cluster carried *Wallaby* (Fig. 2). The *F*_ST_ value of 0.55 between the two clusters indicates a very high level of differentiation. The presence of cheese strains in both clusters indicates that there are major genetic differences between industrial strains that probably predate the domestication of this species for cheese production, given the high level of divergence. The strains in the two clusters also differed in terms of their mating types: the A cluster contained mostly MAT1-2 (91%) strains, whereas the B cluster contained mostly MAT1-1 strains (82%). Overall, the ratio of MAT1-1 to MAT1-2 strains was balanced, with no significant deviation from a 1:1 ratio (*χ*^2^ = 0.27; df = 1; *P *=* *0.6), whereas there was a significant departure from the 1:1 ratio within each cluster (*χ*^2^ = 56.9; df = 1; *P *<* *0.001).

**Figure 3:**
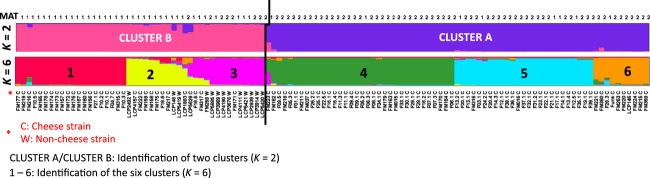
Population structure of *Penicillium roqueforti*. The structure has been inferred by STRUCTURE for *K* = 2 and *K* = 6 (see Figure S2 for the barplots corresponding to other *K* values). The STRUCTURE program could form well-defined clusters up to *K* = 6, indicating the existence of six genetically differentiated clusters. The strongest structure appeared at *K* = 2, with strains split according to a genomic island called *Wallaby,* previously shown to have been horizontally transferred between several *Penicillium* cheese species. CLUSTER A/CLUSTER B: identification of two clusters (*K* = 2); 1–6: identification of the six clusters (*K* = 6).

We investigated the population structure of *P. roqueforti* further, with the STRUCTURE program. STRUCTURE yielded well-defined clusters at *K* values of up to 6 (Fig. 3 and Figure S2), indicating the existence of six genetically differentiated clusters. For the values of *K* of 7 and above, each new cluster included only admixed genotypes, indicating a lack of further structure. At *K* = 2, the structure identified corresponded precisely to that in the splitstree, with the strains separated according to the presence/absence of *Wallaby*. The deltaK value confirmed that this split was the strongest structure in the data set (Figure S3). At *K* = 6, the two clusters defined previously were each split into three well-delimited populations, with some hybrids between populations 2 and 3. Within cluster B, most of the cheese strains (20 of the 32) clustered in population 1, whereas most noncheese strains (10 of the 13) were included in population 3. Moreover, all the cheese strains of population 1 were MAT1-1 strains, whereas population 3 included both mating types, in equal proportions (seven strains of each). Population 2 included mostly MAT1-1 strains from diverse environments (10 MAT1-1 and 1 MAT1-2 strains). No other obvious features, such as geographic origin or type of cheese, could account for the observed population structure.

We performed factorial correspondence analysis to obtain further support for the existence of the six populations from a method assuming no particular population model. The analysis including all 114 strains and 12 loci (the 11 microsatellite markers and the MAT locus) confirmed the differentiation of the three populations identified within cluster B, as these populations did not overlap (Figure S4a, with 12.97% and 9.24% of the variance explained by axes 1 and 2, respectively). Within cluster A, the three populations overlapped when we considered the entire data set and the first two axes (Figure S4a). In an analysis including only the 69 cheese strains belonging to cluster A, it was possible to visualize the differentiation of the three populations identified above by STRUCTURE (13.77% and 12.12% of the variance explained for axes 1 and 2, respectively; Figure S4b).

The existence of six genetically divergent populations was further confirmed by the calculation of *F*_ST_. Within cluster A, including only cheese strains, fixation indices (*F*_ST_) were systematically greater than 0.4, confirming the very high degree of genetic differentiation between the three populations (Table S3). Within cluster B, *F*_ST_ values were greater than 0.5 between populations 1 and 2, and lower (of 0.35) between populations 2 and 3 (Table S3), as expected given the STRUCTURE bar plots indicating the presence of hybrids.

### Recombination footprints in populations

We then investigated the footprints of sex in our population data set. We first performed a split decomposition analysis, showing recombination events as reticulations. SplitsTree analysis yielded a network, with some reticulations but also with long branches (Fig. 2). This pattern is indicative of pervasive clonality with occasional recombination events, within the clusters in particular, and much less frequently between clusters.

When we considered only two genetic groups (*K* = 2), linkage disequilibrium was significant within both clusters, probably due to a Wahlund effect resulting from the genetic structure (Table S4). When six populations were considered, significant linkage disequilibrium was observed only for population 3 within cluster B, but not for the other five populations. Thus, linkage disequilibrium analyses were consistent with the separation of the strains into six groups and the occurrence of recombination within populations.

## Discussion

### Occurrence of sex and recombination

We report here, for the first time, the production of all the typical sexual structures of ascomycete fungi, including ascogonia, cleisthothecia, asci and recombinant ascospores, by the ascomycete *P. roqueforti,* as well as a method for recovering ascospores. Sexual structures were produced only on oatmeal agar medium supplemented with biotin, as reported for *P. chrysogenum,* a close relative of *P. roqueforti* (Böhm et al. 2012). This indicates that this protocol may be largely useful for inducing sex in a wide range of related species including several of industrial importance, such as *P. camemberti*.

Sexual fruiting bodies were abundant in almost all the crosses, regardless of the presence or absence of the *Wallaby* horizontally transferred region, the origin of the strain and its mate. Thus, such structures were observed even in crosses between strains from clusters A and B. Nevertheless, we found mature ascospores in only one of the 16 crosses, in which both parental strains were isolated from noncheese environments (stewed fruit and wood in France) and belonged to population 3 of cluster B. This may reflect strain variability in the time or medium required for ascospore development, or lower levels of fertility in domesticated clonal strains, as suggested for other ascomycetes, such as *A. fumigatus* or *P. chrysogenum* (Dyer and Paoletti 2005; O'Gorman et al. 2009; Böhm et al. 2012; Swilaiman et al. 2013). Further development of the protocol may thus be needed to produce ascospores from cheese strains, but the presence of cleisthothecia in most crosses is promising. No sexual structures were observed on the goat-cheese medium, a nutrient-rich environment. This finding is consistent with theoretical expectations and observations that sexual reproduction is often induced in harsh environments rather than in rich, stable medium (Burt et al. 1996; Goddard et al. 2005; Schoustra et al. 2010). This may have been selected for increasing the speed of adaptation to new environments and/or for the production of sexual structures that are often resistant (Houbraken et al. 2008).

Footprints of recombination were also found in our collection of populations. In particular, in A cluster, which included only cheese strains, none of the three populations identified by STRUCTURE displayed significant linkage disequilibrium and reticulation was observed in the SplitsTree network, indicating the occurrence of recombination between cheese strains. The RIP-like footprints detected in the reference sequenced genome FM164, from a cheese strain carrying *Wallaby* (Cheeseman et al. 2014), and the finding of both mating types in both clusters provide further support for this hypothesis. These footprints of recombination may result from events predating the domestication of the species, which began around the six millennium BC (Salque et al. 2013). Alternatively, recombinant strains from other environments may be regularly introduced in the cheese-making industry. This hypothesis is plausible because *P. roqueforti* is commonly found in dairy-related environments (such as silage) and even in the caves in which the cheese is left to mature, as it tolerates many environmental variations such as cold temperatures, low oxygen concentrations, alkaline and weak acid preservatives (Samson et al. 2004; Pitt and Hocking 2009). However, the frequencies of mating types were not balanced in the different clusters. This may be a consequence of the rarity of sex events, leading to a decrease in the frequency of one mating type by genetic drift. A very low frequency of sexual recombination is consistent with the shape of the SplitsTree diagram, which displays terminal branches without reticulations.

### Population diversity and structure

In this study, we developed 11 new polymorphic microsatellite markers providing unprecedented insight into the population structure and diversity of the cheese fungus *P. roqueforti*. Our analyses revealed the existence of six highly differentiated populations. Such a high genetic differentiation and the few reticulations present between clades on the splitstree together indicate that gene flow has been limited for long between the clusters.

Some cheese strains appeared to belong to clonal lineages, whereas all noncheese strains carried unique genotypes. Cheese strains were found in five of the six populations but, interestingly, the populations split most clearly into two clusters defined on the basis of the presence or absence of *Wallaby*, a large horizontally transferred region thought to provide a competitive advantage against other micro-organisms in cheese (Cheeseman et al. 2014). After an initial horizontal transfer event in *P. roqueforti, Wallaby* may have spread rapidly within populations that were still recombining (i.e. within cluster A), provided that it conferred an advantage. This further indicates that recombination is no longer occurring between clusters A and B. The clusters also appeared to harbour different frequencies of MAT alleles, which are probably due to the lack of gene flow between clusters associated with drift within clusters, because of infrequent sexual reproduction.

The very high level of differentiation suggests that cheese strains in the different clusters may have different metabolic properties, generating different aromas or flavours, for example. It will be interesting to investigate this aspect further, to improve our understanding of the differences between types of blue cheeses and for the development of new flavours. This differentiation may also help to ensure food safety, by facilitating the tracking of strains and the determination of cheese origin.

## Conclusion

We were thus able to induce a sexual cycle under laboratory conditions in the cheese species *P. roqueforti*, for which no sexual structure had ever been observed. This is of great industrial and fundamental importance, as it could facilitate the development of a transformation method for this species, the creation of novel phenotypes by recombination and the purging of deleterious mutations accumulated during clonal propagation. We also provided evidence of a high degree of genetic diversity within the species, and even within cheese strains, with the existence of six populations, probably predating the development of cheese-making, given the high level of differentiation observed. Screening for phenotypic and metabolic differences between these populations could potentially guide future development strategies.

## Data archiving statement

Raw data for this manuscript are available from the Dryad Digital Repository: http://dx.doi.org/10.5061/dryad.t53k5.

## Supporting Information

Figure S1 Neighbour-joining network showing the genetic clustering in *Penicillium roqueforti* according to the presence or absence of the *‘Wallaby’* genomic island, previously shown to have been horizontally transferred between several *Penicillium* cheese species.

Figure S2 Coefficients of membership in the various gene pools inferred by the STRUCTURE program, based on the 11 polymorphic microsatellite markers for all the 114 *Penicillium roqueforti* strains, from *K* = 2 to *K* = 9.

Figure S3 Implementation of the Evanno method for detecting the number of *K* groups for which the subsequent increase in *K* yield less information.

Figure S4 Factorial Correspondence Analyses (FCA) illustrating the differentiation of the six populations of *Penicilium roqueforti*.

Table S1 List of the 114 isolates of *Penicillium roqueforti* used in this study.

Table S2 Names, motif, primer sequences and genome localization of the 11 microsatellites loci used in this study.

Table S3 Fixation indexes (*F*_ST_) between pairs of the *six Penicillium roqueforti* populations defined as in the Figure 2.

Table S4 Linkage disequilibrium within the two main clusters, A and B, defined for *K* = 2 (see Figure 2), and within the six populations (*K* = 6, Figure 2) of *Penicillium roqueforti*.
