# Supplementary figures and images for: Induction of sexual reproduction and genetic diversity in the cheese fungus Penicillium roqueforti
Source: Evol Appl. 2014 Mar 20;7(4):433–41. doi: 10.1111/eva.12140 (PMC4001442; doi:10.1111/eva.12140)

## CLUSTER B

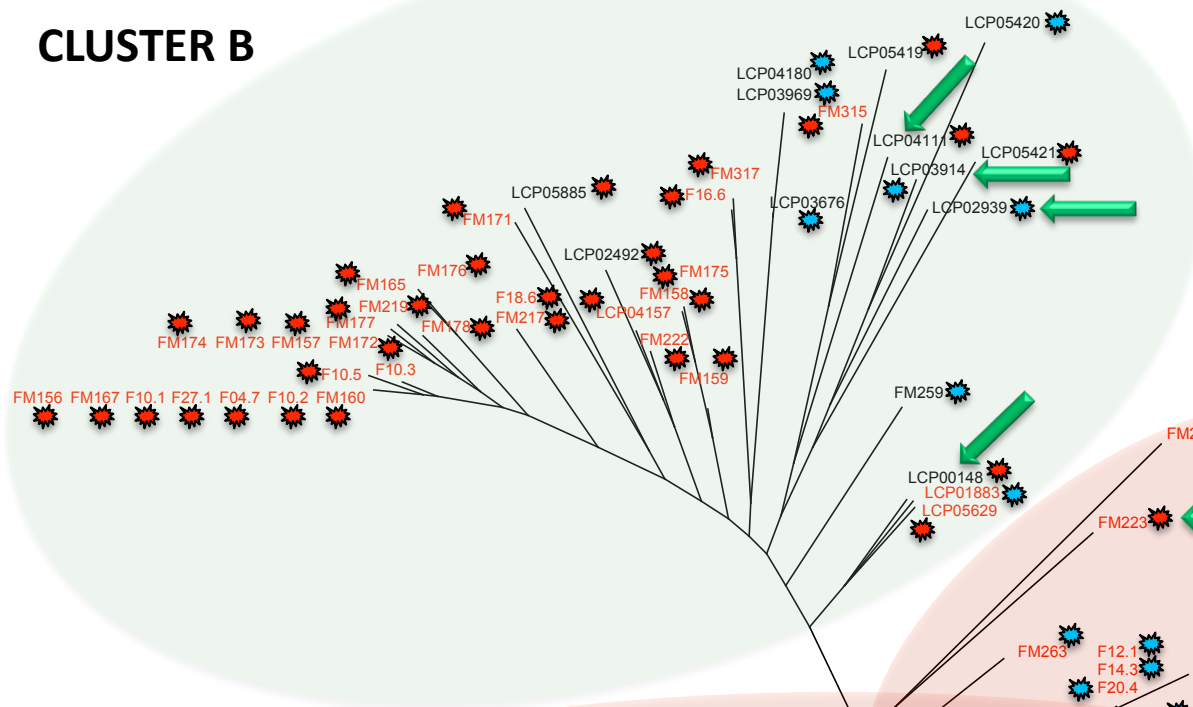

**Cheese strains**  
**Non-cheese strains**  
**Strains used for crosses**

- ★ MAT1-1
- ★ MAT1-2

0.1

## CLUSTER A

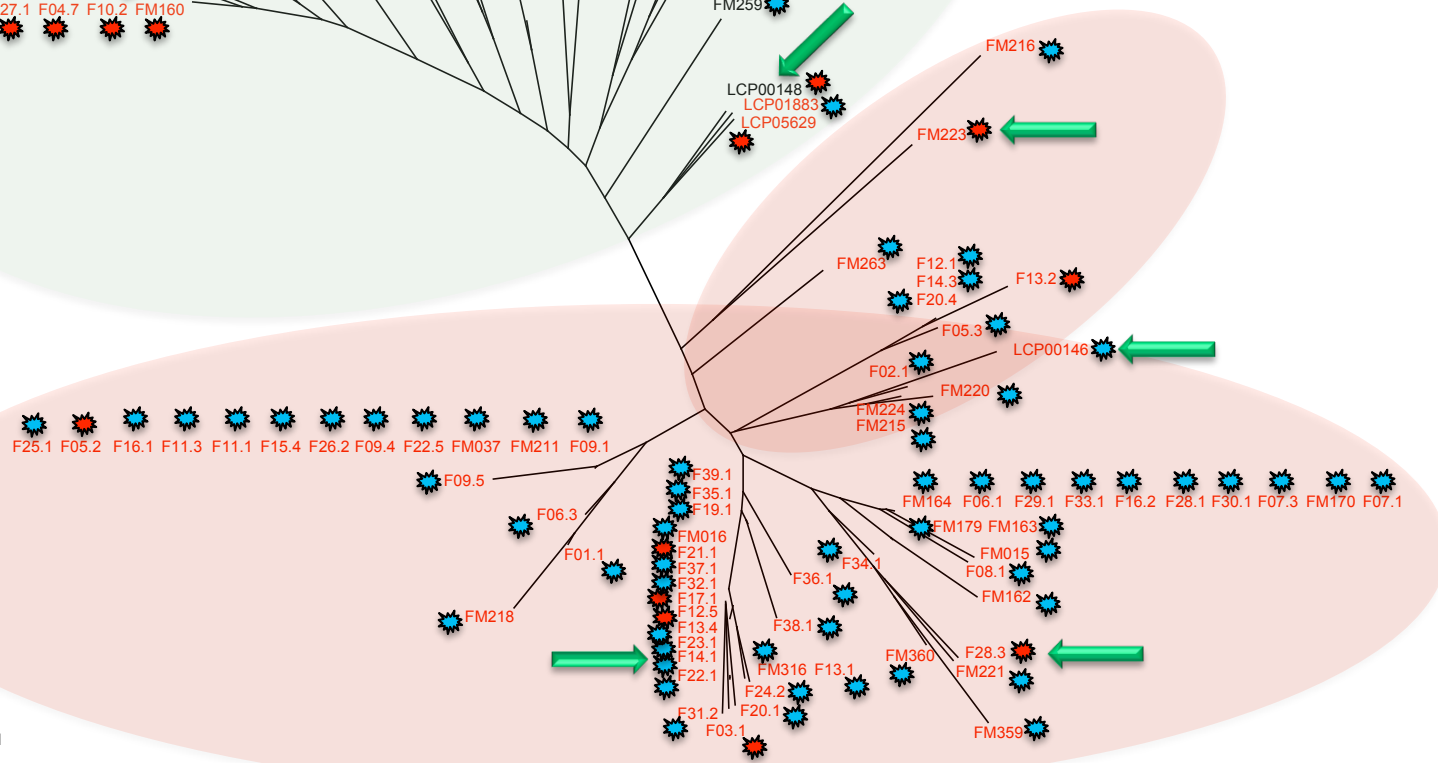

Supplement: Figure S1 — Neighbour-joining network showing the genetic clustering in Penicillium roqueforti according to the presence or absence of the ‘Wallaby’ genomic island, previously shown to have been horizontally transferred between several Penicillium cheese species. [file eva0007-0433-sd1.pdf]

$$\text{DeltaK} = \text{mean}(|L''(K)|) / \text{sd}(L(K))$$

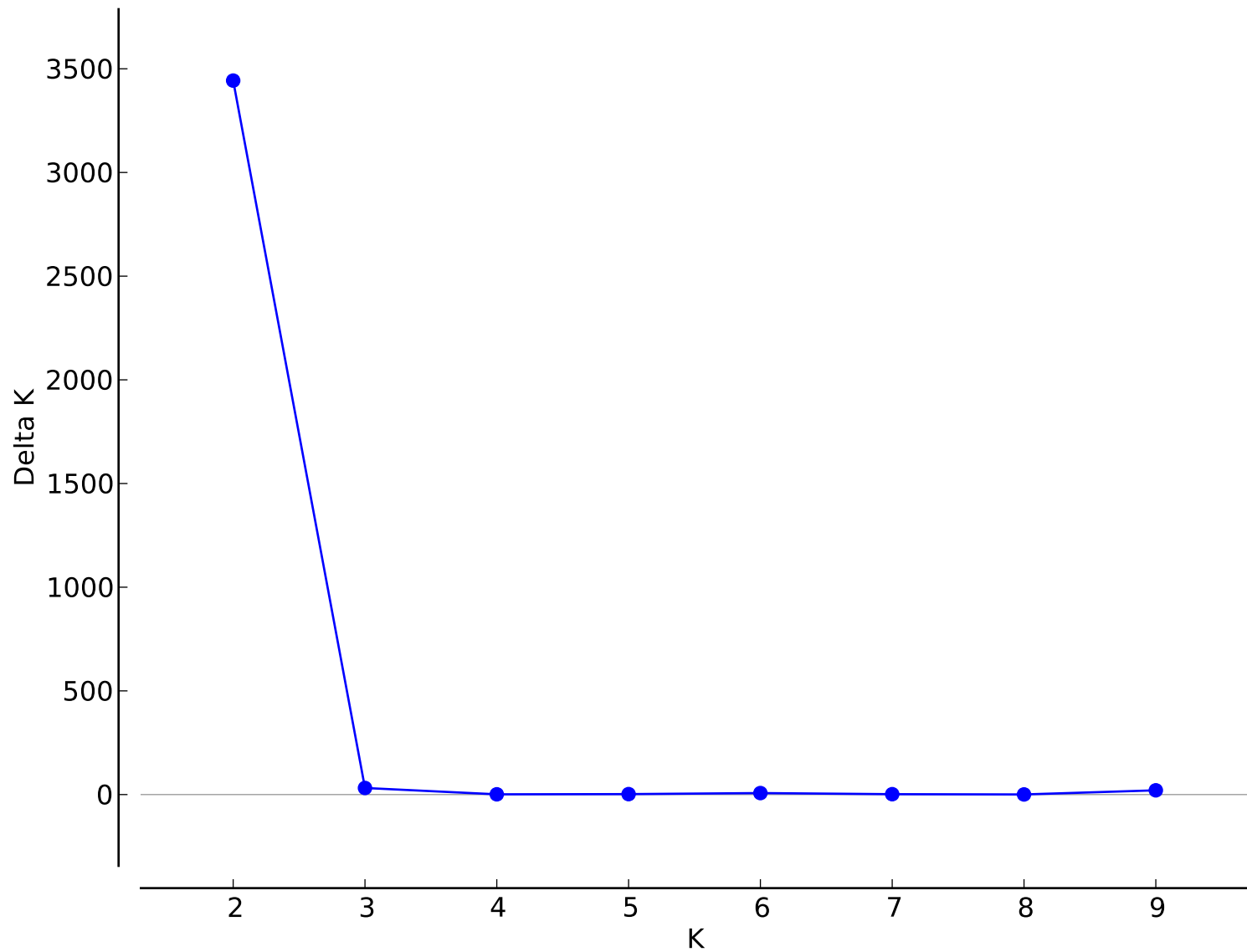

Supplement: Figure S3 — Implementation of the Evanno method for detecting the number of K groups for which the subsequent increase in K yield less information. [file eva0007-0433-sd3.pdf]
