# Supplementary material for: Induction of sexual reproduction and genetic diversity in the cheese fungus Penicillium roqueforti
Source: Evol Appl. 2014 Mar 20;7(4):433–41. doi: 10.1111/eva.12140 (PMC4001442; doi:10.1111/eva.12140)
Supplement: Figure S4 — Factorial Correspondence Analyses (FCA) illustrating the differentiation of the six populations of Penicilium roqueforti. [file eva0007-0433-sd4.pdf]

a) 114 taxa

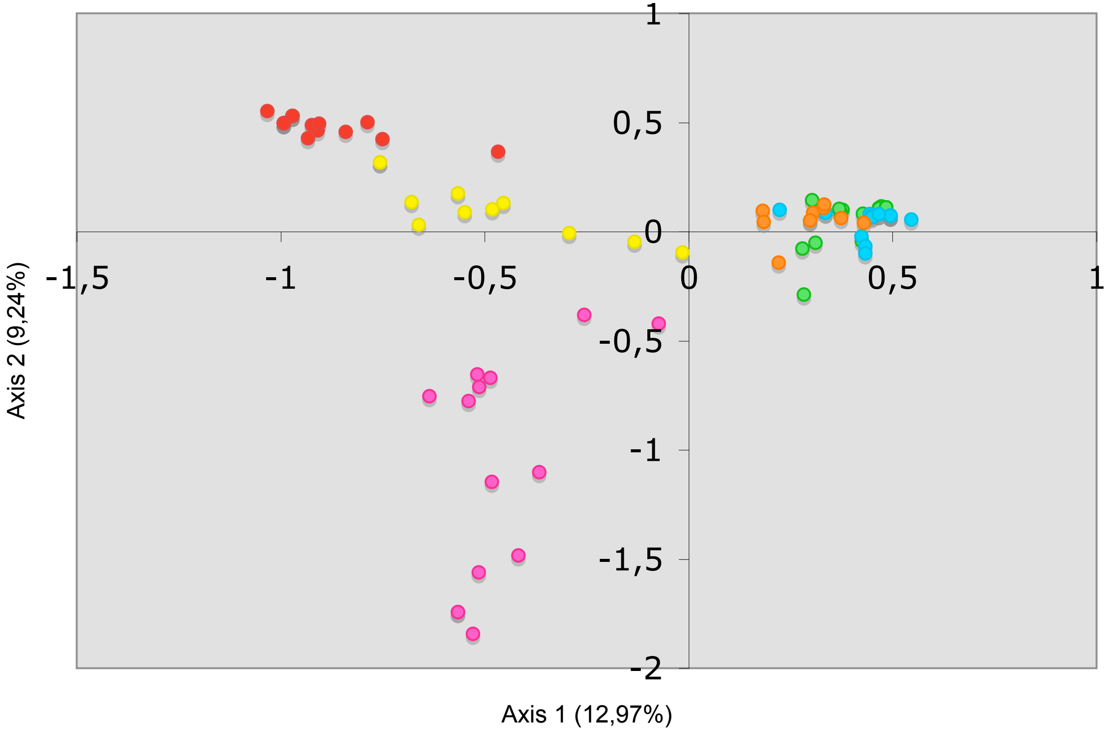

- Population 1
- Population 2
- Population 3
- Population 4
- Population 5
- Population 6

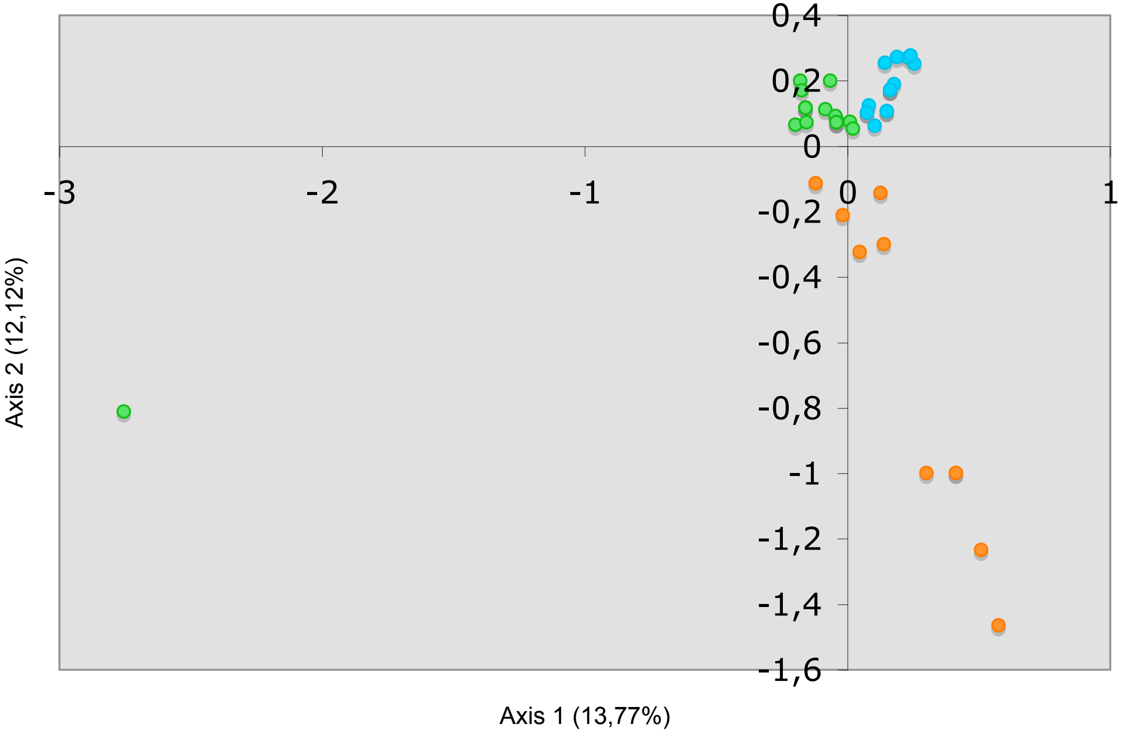

b) 69 taxa (only cluster A included)
