# Supplementary material for: Induction of sexual reproduction and genetic diversity in the cheese fungus Penicillium roqueforti
Source: Evol Appl. 2014 Mar 20;7(4):433–41. doi: 10.1111/eva.12140 (PMC4001442; doi:10.1111/eva.12140)
Supplement: Table S1 — List of the 114 isolates of Penicillium roqueforti used in this study. [file eva0007-0433-sd5.pdf]

	Isolate number		Substrate	Origin	MAT 1-1	MAT 1-2	Wallaby
FM collection	FM 015		Cheese environment	France	-	+	+
	FM016		Cheese environment	France	-	+	+
	FM 037		Cheese environment	France	-	+	+
	FM 156		Cheese environment	France	+	-	-
	FM 157		Cheese environment	France	+	-	-
	FM 158		Cheese environment	France	+	-	-
	FM 159		Cheese environment	France	+	-	-
	FM 160		Cheese environment	France	+	-	-
	FM 162		Cheese environment	France	-	+	+
	FM 163		Cheese environment	France	-	+	+
	FM 164		Gorgonzola cheese	France	-	+	+
	FM 165		Cheese environment	France	+	-	-
	FM 167		Cheese environment	France	+	-	-
	FM 170		Cheese environment	France	-	+	+
	FM 171		Cheese environment	France	+	-	-
	FM 172		Cheese environment	France	+	-	-
	FM 173		Cheese environment	France	+	-	-
	FM 174		Cheese environment	France	+	-	-
	FM 175		Cheese environment	France	+	-	-
	FM 176		Cheese environment	France	+	-	-
	FM 177		Cheese environment	France	+	-	-
	FM 178		Cheese environment	France	+	-	-
	FM 179		Cheese environment	France	-	+	+
	FM 211		Cheese environment	France	-	+	+
	FM 215		Cheese environment	France	-	+	+
	FM 216		Cheese environment	France	+	-	-
	FM 217		Cheese environment	France	+	-	-
	FM 218		Cheese environment	France	-	+	+
	FM 219		Cheese environment	France	+	-	-
	FM 220		Cheese environment	France	-	+	+
	FM 221		Cheese environment	France	-	+	+
	FM 222		Cheese environment	France	+	-	-
	FM 223		Cheese environment	France	+	-	+
	FM 224		Cheese environment	France	-	+	+
	FM 259		Silage	France	-	+	-
	FM 263		Cheese environment	France	-	+	+
	FM 315		Cheese environment	France	+	-	-
	FM 316		Cheese environment	France	-	+	+
	FM 317		Cheese environment	France	+	-	-
	FM 359		Gorgonzola cheese	France	-	+	+
	FM 360		Gorgonzola cheese	France	-	+	+
Public collections	LCP 00146		Roquefort cheese	France	-	+	+
	LCP 00148		Brewery atmosphere	Unknown	+	-	-
	LCP 01883		Cheese	Unknown	-	+	-
	LCP 02492		Unknown	Unknown	+	-	-
	LCP 02939		Packing brioche	France	-	+	-
	LCP 03676		Stewed fruit	France	-	+	-
	LCP 03914		Stewed fruit	France	-	+	-
	LCP 03969		Stewed fruit	France	-	+	-
	LCP 04111		Wood	France	+	-	-
	LCP 04157	IMI 024313T	Roquefort cheese	Unknown	+	-	-
	LCP 04180		Strawberry sorbet	France	-	+	-
	LCP 05419		Fridge inner wall	France	+	-	-
	LCP 05420		Fridge inner wall	France	-	+	-
	LCP 05421		Fridge inner wall	France	+	-	-
	LCP 05885		Silage	France	+	-	-
	LCP 05629	CBS 449.78	Cheddar cheese	Unknown	-	+	-
	F01.1		Blue cheese	Canada	-	+	+

	Isolate number	Substrate	Origin	MAT 1-1	MAT 1-2	Wallaby
LUBEM Brest collection (directly isolated from various blue cheeses)	F02.1	Blue cheese	Canada	-	+	+
	F03.1	Blue cheese	Canada	-	+	+
	F04.7	Blue cheese	Canada	+	-	-
	F05.2	Fourme d'Ambert	France	+	-	+
	F05.3	Fourme d'Ambert	France	-	+	+
	F06.1	Gorgonzola	Italy	-	+	+
	F06.3	Gorgonzola	Italy	-	+	+
	F07.1	Gorgonzola	Italy	-	+	-
	F07.3	Gorgonzola	Italy	-	+	+
	F08.1	Gorgonzola	Italy	-	+	+
	F09.1	Fourme d'Ambert	France	-	+	+
	F09.4	Fourme d'Ambert	France	-	+	+
	F09.5	Fourme d'Ambert	France	-	+	-
	F10.1	Bleu d'Auvergne	France	+	-	-
	F10.2	Bleu d'Auvergne	France	+	-	-
	F10.3	Bleu d'Auvergne	France	+	-	-
	F10.5	Bleu d'Auvergne	France	+	-	-
	F11.1	Carré Aurillac	France	-	+	+
	F11.3	Carré Aurillac	France	-	+	+
	F12.1	Pigme	France	-	+	+
	F12.5	Pigme	France	+	-	+
	F13.1	Carré Auvergne	France	-	+	-
	F13.2	Carré Auvergne	France	+	-	+
	F13.4	Carré Auvergne	France	-	+	+
	F14.1	Gorgonzola	Argentina	-	+	+
	F14.3	Gorgonzola	Argentina	-	+	+
	F15.4	Blue cheese	Brazil	-	+	+
	F16.1	Picon Hoja	Spain	-	+	+
	F16.2	Picon Hoja	Spain	-	+	+
	F16.6	Picon Hoja	Spain	+	-	-
	F17.1	Peña Santa	Spain	+	-	+
	F18.6	Tresviso	Spain	+	-	-
	F19.1	Peral	Spain	-	+	+
	F20.1	Cabrales	Spain	-	+	+
	F20.4	Cabrales	Spain	-	+	+
	F21.1	Blue cheese	Spain	+	-	+
	F22.1	Blue cheese	Netherlands	-	+	+
	F22.5	Blue cheese	Netherlands	-	+	-
	F23.1	Blue cheese	Netherlands	-	+	+
	F24.2	Blue cheese	Netherlands	-	+	+
	F25.1	Blue cheese	Netherlands	-	+	+
	F26.2	Blue cheese	Netherlands	-	+	+
	F27.1	Blue cheese	USA	+	-	-
	F28.1	Blue cheese	Latvia	-	+	+
	F28.3	Blue cheese	Latvia	+	-	+
	F29.1	Blue cheese	Denmark	-	+	+
	F30.1	Blue cheese	Poland	-	+	+
	F31.2	Blue cheese	Latvia	-	+	+
	F32.1	Blue cheese	Denmark	-	+	+
	F33.1	Blue cheese	Germany	-	+	+
	F34.1	Blue cheese	Estonia	-	+	+
	F35.1	Blue cheese	Germany	-	+	+
	F36.1	Blue cheese	Germany	-	+	+
	F37.1	Blue cheese	Germany	-	+	+
	F38.1	Blue cheese	Germany	-	+	+
	F39.1	Blue cheese	Germany	-	+	+
