# Supplementary material for: Induction of sexual reproduction and genetic diversity in the cheese fungus Penicillium roqueforti
Source: Evol Appl. 2014 Mar 20;7(4):433–41. doi: 10.1111/eva.12140 (PMC4001442; doi:10.1111/eva.12140)
Supplement: Table S2 — Names, motif, primer sequences and genome localization of the 11 microsatellites loci used in this study. [file eva0007-0433-sd6.pdf]

Primer name	Repetition pattern	Primer Forward		Primer Reverse	
		Sequence	Position on the reference genome	Sequence	Position on the reference genome
PROQ12	AT	ATCAATGAAACCGCGAAAAG	Proq06 395871 - 395890	GCTCGGGATTATCAGGTTTG	Proq06 396057 - 396038
PROQ13	AT	TGTGCCTATCGGATTTGATG	Proq06 1099272 - 1099291	ACGTCACAGCCAAGTTAGGG	Proq06 1099454 - 1099435
PROQ73	TTC	GAGCACGGCATGAGTCTGTA	Proq03 1674326 - 1674345	CTGACGCATTATCGCTTGAA	Proq03 1674507 - 1674488
PROQ74	TTC	ATGCACACTAACCGGGAAAA	Proq07 451954 - 451973	GCCTGGAATTGAGCCACTTA	Proq07 452109 - 452090
PROQ75	TTC	GCTCGCTTCTGAGATGGACT	Proq16 38357 - 38376	CCATGGCCCTGGTAATAATG	Proq16 38511 - 38492
PROQ77	CTC	ACGACCAGTCGCGTTAATCT	Proq04 1071562 - 1071581	GGTGTGAACAGTTTGCGGTA	Proq04 1071758 - 1071739
PROQ78	ACT	CGGGTTGCCTTAGGCTCT	Proq03 968614 - 968631	CGCTAATTGGAAATGCTGAC	Proq03 968812 - 968793
PROQ80	CTG	AGACTTATCGCCCGTGACAG	Proq07 268923 - 268942	TGAGCACTCGGTTGTACCAG	Proq07 269115 - 269096
PROQ81	CTG	GCGGGAGTTAGAGGACGAAT	Proq12 110107 - 110126	GTGCCAGACAACAACAGCAG	Proq12 110291 - 110272
PROQ88	AAC	CAGGAACAGCAGCAATTTCA	Proq07 400257 - 400276	GCGATTGTCTCTCGAAGGTG	Proq07 400439 - 400420
PROQ93	AAG	CGGTCTATGCAATAAACAAGC	Proq12 420089 - 420109	CCTCGCTCCTTTTTCCTCTC	Proq12 420264 - 420245
