# Supplementary material for: Induction of sexual reproduction and genetic diversity in the cheese fungus Penicillium roqueforti
Source: Evol Appl. 2014 Mar 20;7(4):433–41. doi: 10.1111/eva.12140 (PMC4001442; doi:10.1111/eva.12140)
Supplement: Table S3 — Fixation indexes (FST) between pairs of the six Penicillium roqueforti populations defined as in the Figure 2. [file eva0007-0433-sd7.pdf]

		CLUSTER B			CLUSTER A	
		Population 1	Population 2	Population 3	Population 4	Population 5
CLUSTER B	Population 2	0.5950				
	Population 3	0.5951	0.3450			
CLUSTER A	Population 4	0.8336	0.6991	0.6024		
	Population 5	0.9013	0.7869	0.6739	0.5127	
	Population 6	0.7945	0.5798	0.4156	0.4001	0.5314

Genetic differentiation between cluster A and cluster B

Genetic differentiation within cluster A

Genetic differentiation within cluster B
