# Supplementary material for: Induction of sexual reproduction and genetic diversity in the cheese fungus Penicillium roqueforti
Source: Evol Appl. 2014 Mar 20;7(4):433–41. doi: 10.1111/eva.12140 (PMC4001442; doi:10.1111/eva.12140)
Supplement: Table S4 — Linkage disequilibrium within the two main clusters, A and B, defined for K = 2 (see Figure 2), and within the six populations (K = 6, Figure 2) of Penicillium roqueforti. [file eva0007-0433-sd8.pdf]

K=2

	Couples in linkage disequilibrium		Invalid data	
	Individuals	%	Individuals	%
CLUSTER B	28/66	42.42	21/64	32.81
CLUSTER A	7/66	10.60	25/66	37.88

K = 6

		Couples in linkage disequilibrium		Invalid data	
		Individuals	%	Individuals	%
CLUSTER B	Population 1	1/66	1.55	60/66	90.9
	Population 2	1/66	1.55	45/66	68.18
	Population 3	9/66	13.64	21/66	31.82
CLUSTER A	Population 4	3/66	4.55	38/66	57.58
	Population 5	0/66	0	55/66	83.33
	Population 6	1/66	1.55	40	66.67
