# Supplementary material for: Induction of sexual reproduction and genetic diversity in the cheese fungus Penicillium roqueforti
Source: Evol Appl. 2014 Mar 20;7(4):433–41. doi: 10.1111/eva.12140 (PMC4001442; doi:10.1111/eva.12140)
Supplement: Figure S2 — Coefficients of membership in the various gene pools inferred by the STRUCTURE program, based on the 11 polymorphic microsatellite markers for all the 114 Penicillium roqueforti strains, from K = 2 to K = 9. [file eva0007-0433-sd2.pdf]

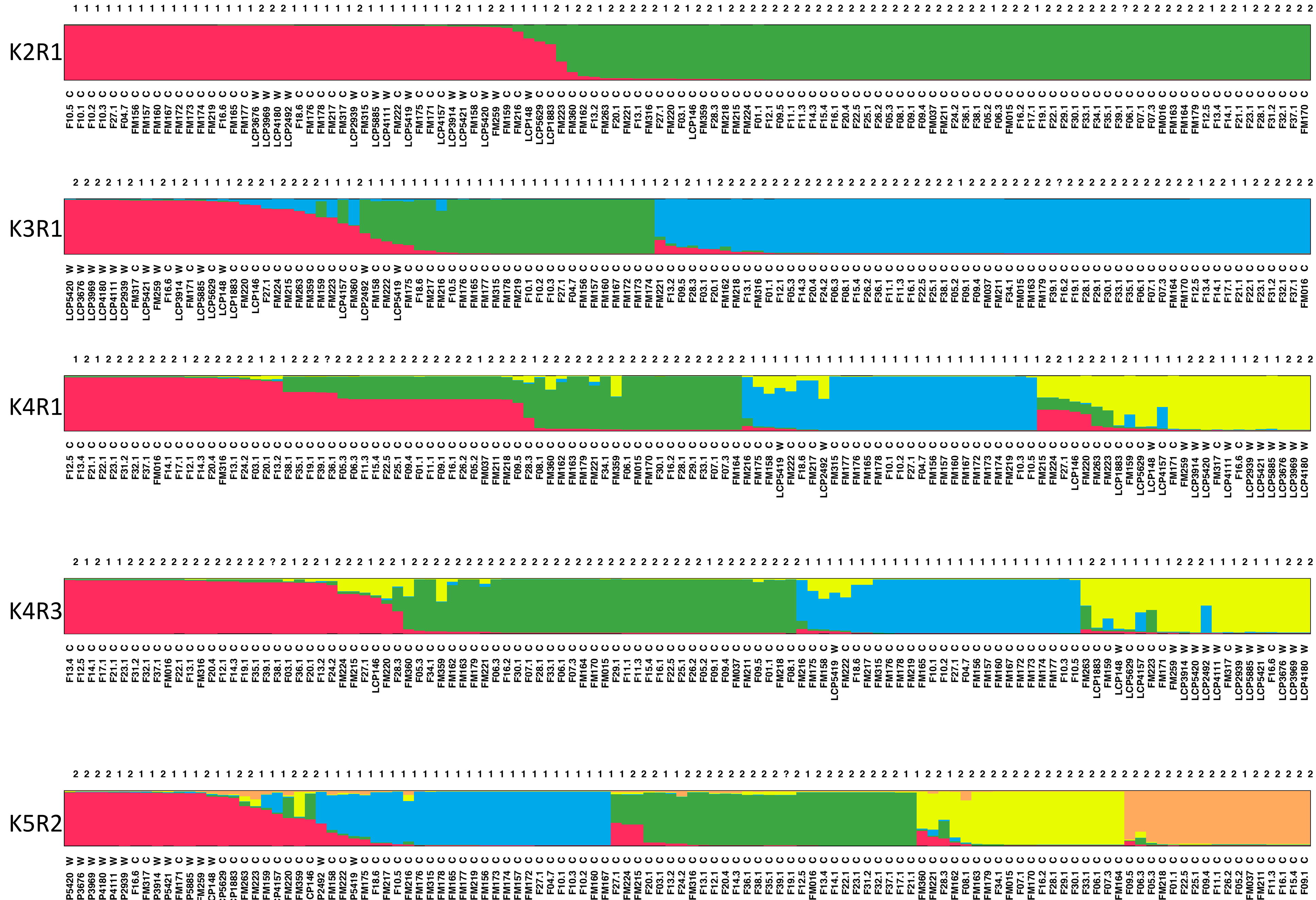
K2R1
K3R1
K4R1
K4R3
K5R2

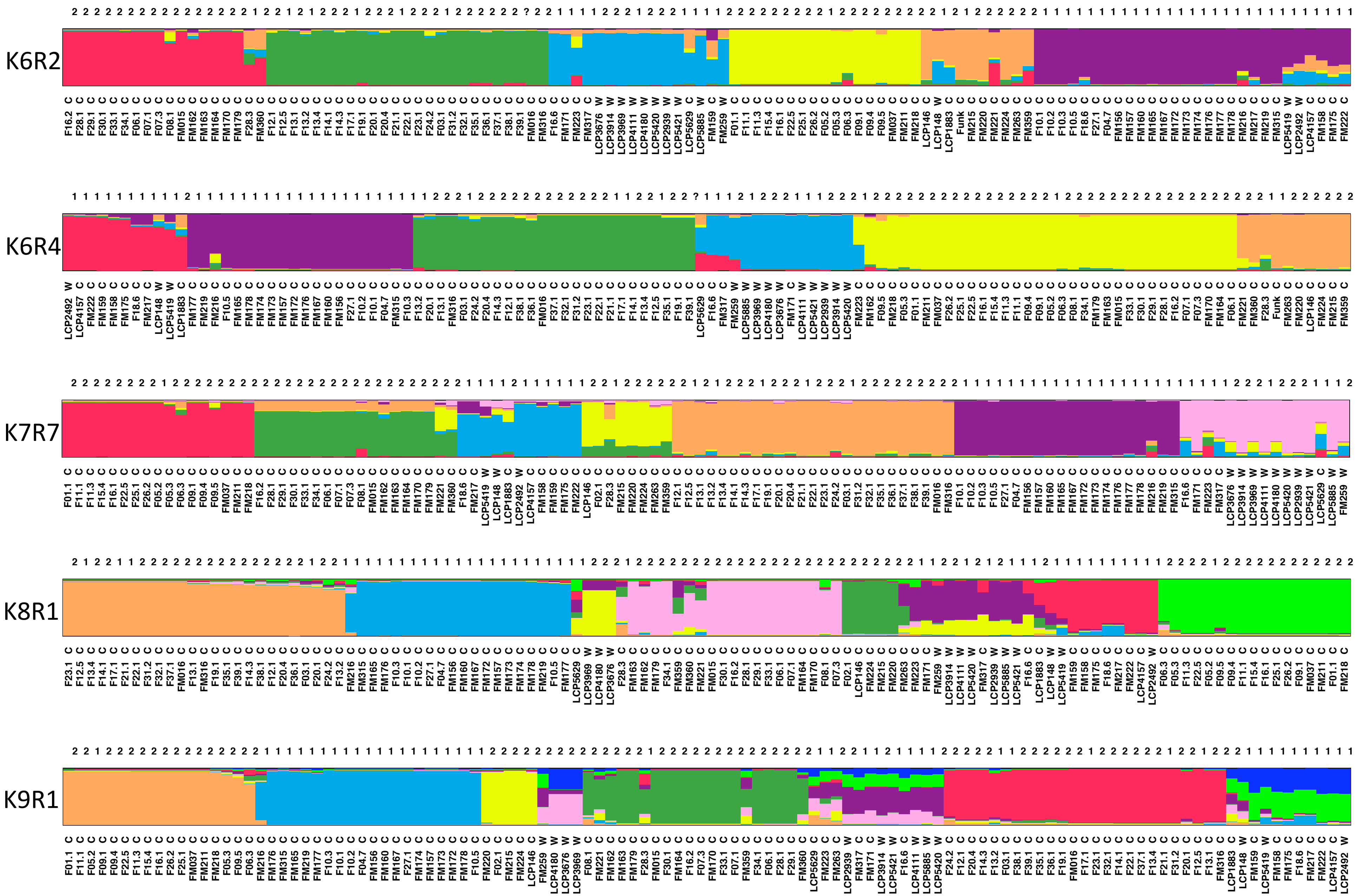
K6R2
K6R4
K7R7
K8R1
K9R1
